# HER2 Protein Overexpression and Gene Amplification in Plasmacytoid Urothelial Carcinoma of the Urinary Bladder

**DOI:** 10.1155/2016/8463731

**Published:** 2016-03-10

**Authors:** Bohyun Kim, Gilhyang Kim, Boram Song, Cheol Lee, Jeong Hwan Park, Kyung Chul Moon

**Affiliations:** ^1^Department of Pathology, Seoul National University College of Medicine, Seoul 03080, Republic of Korea; ^2^Kidney Research Institute, Medical Research Center, Seoul National University College of Medicine, Seoul 03080, Republic of Korea

## Abstract

*Aim*. HER2 overexpression has been reported in a minority of urothelial carcinomas, but little is known about HER2 protein expression and gene alterations in plasmacytoid urothelial carcinoma, a rare and aggressive variant. The aim of this study was to clarify the HER2 status in plasmacytoid urothelial carcinomas.* Methods*. Six cases of plasmacytoid urothelial carcinoma were included, in which we evaluated HER2 protein expression by immunohistochemistry (IHC) and* HER2* gene amplification by fluorescence* in situ* hybridization (FISH).* Results*. The patients' ages ranged from 57 to 83 years (mean age, 71 years). Five patients were male and one was female. The ratio of the plasmacytoid component ranged from 30% to 100% (mean, 77%). HER2 expression score was 3+ in 4 cases, 2+ in one case, and negative in one case. HER2 gene amplification was positive in 3 cases, of which 2 cases showed a 3+ HER2 IHC score but one case was negative for HER2 IHC. Another 2 cases showed equivocal HER2 FISH results, and one remaining case was negative for HER2 FISH.* Conclusion*. Our observation that plasmacytoid urothelial carcinomas frequently demonstrated HER2 protein overexpression provides supporting evidence that HER2 may be a potential therapeutic target for plasmacytoid urothelial carcinoma.

## 1. Introduction

Many histologic variants of urothelial carcinoma have been described [1–3]. Variants of urothelial carcinoma show different prognosis [4]. Among these variants, plasmacytoid urothelial carcinoma is a rare and aggressive variant of urothelial carcinoma and is associated with poor prognosis [5–10].

Human epidermal growth factor receptor type 2 (HER2) is a transmembrane receptor tyrosine kinase, and its coding gene is located on chromosome band 17q21 [11]. HER2 overexpression in cancer is associated with poor prognosis in various cancers [12], and anti-HER2 therapy is well established for HER2 overexpressing breast cancers and gastric cancers [13, 14]. Recently, HER2 protein overexpression and gene amplification have been reported in urothelial carcinomas, and some studies have shown the prognostic significance of HER2 overexpression or gene amplification in urothelial carcinoma [15–18]. As a result, HER2 is being considered as a new therapeutic target for urothelial carcinomas [19, 20].

The frequency of HER2 overexpression or gene amplification in urothelial carcinoma is approximately 10% [15, 19, 21]. Among the known variants of urothelial carcinoma, the micropapillary variant of urothelial carcinoma frequently showed HER2 protein overexpression and HER2 gene amplification [22]. Additionally, HER2 gene amplification in the micropapillary variant of urothelial carcinoma is associated with poor outcome [23]. A recent study also revealed the different incidence of HER2 gene amplification among the variants of urothelial carcinoma [24]. However, little is known about HER2 expression and gene amplification in plasmacytoid urothelial carcinoma. Only one study showed HER2 protein overexpression in 2 cases of plasmacytoid urothelial carcinoma with concurrent micropapillary component [25].

In the present study, we investigated HER2 protein expression and HER2 gene amplification in plasmacytoid urothelial carcinomas.

## 2. Materials and Methods

### 2.1. Case Selection and Tissue Microarray (TMA) Construction

We searched the computerized database of the Department of Pathology, Seoul National University Hospital, and we found six cases that had been diagnosed as plasmacytoid urothelial carcinoma in bladder tumor specimens between 2005 and July 2015. We reviewed the hematoxylin and eosin-stained slides to confirm the adequacy of the diagnosis and various pathologic parameters, including the percentage of plasmacytoid urothelial carcinoma component, the presence of concomitant conventional urothelial carcinoma and other variants, and the TNM stage. We collected the clinical data and pathologic information from the medical records and pathology reports. A TMA block was prepared from formalin-fixed paraffin-embedded tissue blocks (SuperBioChips Laboratories, Seoul, Korea). Two to seven cores containing various tumor areas were obtained from each case. This study was approved by the Institutional Review Board (IRB) of Seoul National University Hospital.

### 2.2. Immunohistochemistry (IHC)

Immunohistochemical staining for HER2 was performed using the HercepTest*™* kit (Dako, Glostrup, Denmark) according to manufacturer's protocols. 4 *μ*m thick sections taken both from original full tumor blocks and the TMA block were used for HER2 IHC. HER2 protein expression was scored as 0, 1+, 2+, and 3+ according to the ASCO/CAP 2013 HER2 test guideline [26]. HER2 IHC 3+ was considered to be HER2-positive, IHC 2+ HER2-equivocal, and IHC 0 and 1+ HER2-negative [26]. Additional immunohistochemical staining for cytokeratin (CK) 7 (clone OV-TL 12/30; Dako), CK20 (clone Ks20.8; Dako), p53 (clone DO7; Dako), p63 (clone 4A4; Ventana), and CD138 (clone 5F7; Novocastra) was also performed on sections taken from the TMA block. All IHC were performed using Ventana Benchmark XT automated staining system (Ventana Medical Systems, Tucson, AZ).

### 2.3. Fluorescence* In Situ* Hybridization (FISH)

HER2 gene amplification was examined by dual-color FISH analysis using PathVysion HER2 DNA Probe Kit (Abbott, Abbott Park, IL) on TMA sections according to the manufacturer's protocols. FISH results were analyzed by counting the fluorescence signals in 20 malignant cells. For each case, the average* HER2* copy number and the ratio of* HER2* signals to chromosome 17 centromere (CEP17) signals were calculated. HER2 positivity by FISH was defined as an average* HER2* copy number ≥6.0 or* HER2*/CEP17 ratio ≥2.0 according to the ASCO/CAP 2013 HER2 test guideline [26]. The cases showing* HER2*/CEP17 ratio <2.0 with average* HER2* copy number ≥4.0 and <6.0 were regarded as HER2-equivocal, and the cases showing* HER2*/CEP17 ratio <2.0 with average* HER2* copy number <4.0 were regarded as HER2-negative.

The results of the HER2 test were considered positive when the tumor specimens showed HER2 IHC 3+ or positive* HER2* gene amplification by FISH.

## 3. Results

### 3.1. Clinical and Pathologic Characteristics of Plasmacytoid Urothelial Carcinomas

**Figure 1:**
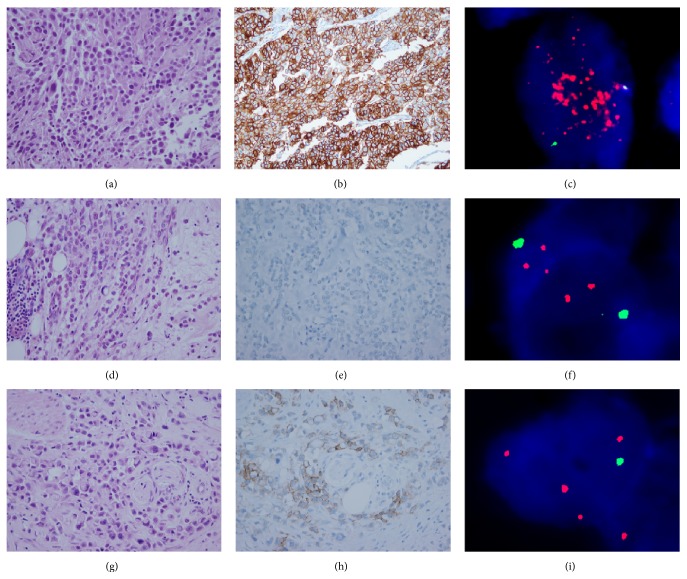
Plasmacytoid component of case 1 (a) showed diffuse 3+ HER2 IHC positivity (b) and* HER2* gene amplification (*HER2*/CEP17 ratio 5.8) by FISH (c). Case 3 (d) was negative for HER2 by IHC (e), but the* HER2* gene was amplified (*HER2*/CEP17 ratio 2.06) (f). Case 4 (g) showed 2+ HER2 by IHC (h) and equivocal* HER2* FISH results (*HER2*/CEP17 ratio 1.81 and average* HER2* gene copy number 4.9) (i).

Six plasmacytoid urothelial carcinoma cases were included in this study, of which 5 were male and one was female.  Table 1 summarizes the clinical and pathologic features of these 6 cases. The mean age was 71 years (range, 57–83 years). The initial presenting symptom was hematuria in 5 cases and urgency in one case. Four patients had TNM stage IV disease, one stage III disease, and one stage I disease. The proportion of the plasmacytoid component ranged from 30% to 100% (mean, 77%) (Figures 1(a), 1(d), and 1(g)). Two cases consisted entirely of plasmacytoid components (Cases 4 and 5), and remaining four cases contained conventional or micropapillary urothelial carcinoma components (Table 1).

### 3.2. HER2 Protein Expression and* HER2* Gene Amplification

The HER2 IHC and FISH results are summarized in Table 2. Four cases showed IHC 3+ by HER2 IHC, and in these four cases, most tumor cells with plasmacytoid morphology showed intense membranous HER2 staining (Figure 1(b)). One case had equivocal HER2 immunostaining (IHC 2+) (Figure 1(h)) and one remaining case did not stain for HER2 (IHC 0) (Figure 1(e)). By* HER2* FISH, three cases showed a* HER2*/CEP17 ratio ≥2.0 (Figures 1(c) and 1(f)). Another two cases had* HER2*/CEP17 ratios <2.0 but with an average* HER2* copy number ≥4.0 and were considered to be equivocal for* HER2* gene amplification. One case (case 3) was negative for HER2 by IHC but FISH demonstrated positive* HER2* gene amplification (Figures 1(h) and 1(i)). Overall, the HER2 test was positive in five cases and equivocal in one case (Table 2). HER2 IHC results of conventional or micropapillary components were identical to those of plasmacytoid components.* HER2* gene amplification of conventional or micropapillary components was positive in 2 cases and negative in remaining two cases (Table 2).

### 3.3. Immunohistochemical Staining

**Figure 2:**
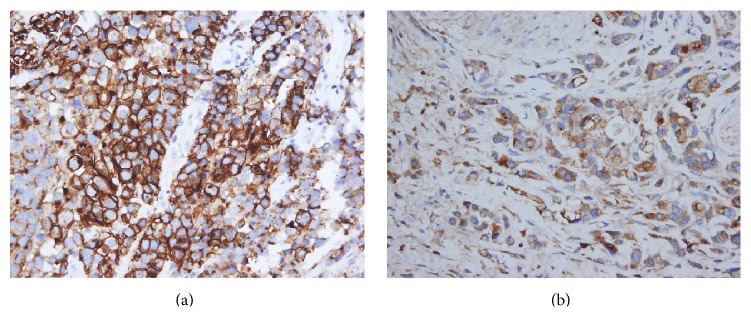
Case 1 (a) and case 4 (b) showed strong diffuse immunopositivity for CD138.

Immunohistochemical staining results are detailed in Table 3. CK7 was positive in five cases, CK20 in six cases, and p63 in four cases. CD138, an immunohistochemical marker for plasma cells, was positive in all six cases (Figure 2). p53 was diffusely positive in one case and focally positive in four other cases.

## 4. Discussion

Urothelial carcinoma is the most common cancer of the urinary bladder. Many histologic variants of urothelial carcinoma have been reported, and these variants showed clinicopathological features distinct from those of conventional urothelial carcinoma [1, 2, 4]. Plasmacytoid urothelial carcinoma is a rare variant of urothelial carcinoma characterized by tumor cells that resemble plasma cells [5, 6].

In the present study, we evaluated the HER2 protein expression and* HER2* gene amplification in six cases of plasmacytoid urothelial carcinoma. Our study revealed that plasmacytoid urothelial carcinoma frequently showed HER2 protein overexpression and* HER2* gene amplification. Five cases out of six were considered to be HER2-test-positive and one was considered as equivocal according to the ASCO/CAP 2013 HER2 test guideline. Little is known about the HER2 status in plasmacytoid urothelial carcinoma. Only one case report showed HER2 protein expression by IHC in two plasmacytoid urothelial carcinoma cases, of which one case was HER2 IHC 3+ and the other one 2+ [25]. There has been no study about* HER2* gene amplification in plasmacytoid urothelial carcinoma. To the best of our knowledge, our study is the first to demonstrate that the* HER2* gene is frequently amplified in plasmacytoid urothelial carcinoma.

*HER2* gene amplification is found in a small subset of urothelial carcinomas and is related to poor prognosis [15, 16]. Among the variants of urothelial carcinoma, the micropapillary variant of urothelial carcinoma showed a higher incidence of* HER2* gene amplification and HER2 protein overexpression than conventional urothelial carcinoma. Ching et al. revealed that 68% of micropapillary urothelial carcinoma (13/19) overexpressed HER2 protein (2+ to 3+ by HER2 IHC), and 42% (8/19) showed gene amplification [22]. Although the number of cases was limited, our study showed that the majority of cases of plasmacytoid urothelial carcinoma were also positive in the HER2 test.

HER2 overexpression and gene amplification have been known to be related to the aggressive behavior of various cancers including urothelial carcinoma [12, 15, 16, 18]. Micropapillary urothelial carcinoma is also an aggressive variant, and HER2 gene amplification may be related to the poor prognosis of this variant [23]. In this regard, HER2 overexpression and gene amplification in plasmacytoid urothelial carcinoma may be associated with the aggressive behavior of this tumor.

In this study, two cases (cases 3 and 5) showed discrepancies between the results of HER2 IHC and FISH. Some previous studies also showed the discrepancies between HER2 IHC and FISH in some urothelial carcinoma cases [22, 23]. Furthermore a previous study described that there was no strong association between HER2 protein overexpression and gene amplification in contrast to breast cancer [27]. This study also revealed the discrepancies between the results of HER2 IHC and FISH in two out of six plasmacytoid urothelial carcinomas. Further study will be needed to elucidate these discrepancies in urothelial carcinoma.

HER2 is a well-established therapeutic target in some cancers characterized by HER2 protein overexpression or gene amplification [13, 14]. Targeted therapy against HER2 has been attempted in patients with urothelial carcinoma showing HER2 gene amplification [20, 28]. Plasmacytoid urothelial carcinoma is a very aggressive variant [4]. Although neoadjuvant chemotherapy for this tumor led to improvement of the pathological stage of patients in a study, the outcome was dismal [8]. The results of our study suggested that HER2 may be a good candidate for targeted therapy of plasmacytoid urothelial carcinoma.

CD138 is known to be a marker for plasma cells, and variable expression of CD138 in plasmacytoid urothelial carcinoma has been reported in previous studies [5, 6, 10, 25]. CD138 is also expressed in urothelial carcinoma* in situ*, conventional urothelial carcinoma, and even normal urothelium [29, 30]. Our study confirmed the expression of CD138 in plasmacytoid urothelial carcinoma, and the incidence was 100% (6/6 cases).

## 5. Conclusion

In summary, our study demonstrated the frequent occurrence of HER2 protein overexpression and gene amplification in plasmacytoid urothelial carcinomas. This result provides supporting evidence that HER2 may be a potential therapeutic target for the control of this aggressive tumor.

## Figures and Tables

**Table 1:** Clinicopathologic characteristics of plasmacytoid urothelial carcinomas.

Case	Age/sex	Presenting symptoms	Operation	TNM stage	Plasmacytoid component	Other components	Follow-up status
1	64/M	Hematuria	TUR	IV	30%	CONV	LOF, after 16 mo
2	83/M	Hematuria	RC	IV	80%	CONV, MP	DOD, 28 mo
3	69/M	Urgency	RC	III	80%	CONV	DOD, 10 mo
4	73/M	Hematuria	RC	IV	100%	None	LOF, after 3 mo
5	57/M	Hematuria	RC	IV	100%	None	AWD, 19 mo
6	82/F	Hematuria	TUR	I	70%	CONV	AWD, 1 mo

AWD indicates alive with disease; CONV, conventional type; DOD, died of disease; F, female; LOF, loss of follow-up; M, male; MP, micropapillary type; RC, radical cystectomy; TUR, transurethral resection.

**Table 2:** HER2 IHC and FISH results.

Case	Component	HER2 IHC	*HER2* gene amplification	*HER2*/CEP17 ratio	Average *HER2* copy number
1	P	Positive (3+)	Positive	5.8	20.0
	CONV	Positive (3+)	Positive	3.03	9.0

2	P	Positive (3+)	Positive	3.61	9.8
	MP	Positive (3+)	Positive	2.2	9.0
	CONV	Positive (3+)	Positive	2.13	7.5

3	P	Negative (0)	Positive	2.06	4.9
	CONV	Negative (0)	Negative	1.27	2.0

4	P	Equivocal (2+)	Equivocal	1.81	4.9

5	P	Positive (3+)	Negative	1.68	3.5

6	P	Positive (3+)	Equivocal	1.61	4.5
	CONV	Positive (3+)	Negative	1.07	3.2

P, plasmacytoid component; CONV, conventional component; MP, micropapillary component.

**Table 3:** Immunohistochemical staining results.

Case	p63	CK7	CK20	p53	CD138
1	P	P	P	Focal strong P	P
2	P	P	P	Negative	P
3	N	N	Focal P	Focal moderate P	P
4	N	P	Focal P	Focal strong P	P
5	P	P	P	Diffuse strong P	P
6	P	P	P	Focal moderate P	P

P indicates positive; N, negative.
